# A Conceptual Review of Positive Teacher Interpersonal Communication Behaviors in the Instructional Context

**DOI:** 10.3389/fpsyg.2021.708490

**Published:** 2021-07-15

**Authors:** Fei Xie, Ali Derakhshan

**Affiliations:** ^1^School of College English Teaching and Research, Henan University, Zhengzhou, China; ^2^Department of English Language and Literature, Faculty of Humanities and Social Sciences, Golestan University, Gorgan, Iran

**Keywords:** conceptual review, teacher interpersonal communication, positive psychology, instructional communication, positive teacher–student relationships, student-related academic outcomes

## Abstract

Following the recent special issue in Frontiers in Psychology, entitled “*The Role of Teacher Interpersonal Variables in Students’ Academic Engagement, Success, and Motivation*,” calling educational researchers worldwide to examine different teacher interpersonal communication behaviors that contribute to student-related academic outcomes, this conceptual review article is written to familiarize educational researchers, teachers, and students with main concepts in instructional communication and their role as the main pillar of successful teaching and learning processes. To this aim, by drawing on the positive psychology movement and the rhetorical and relational goal theory in instructional communication, we argue that positive teacher interpersonal communication behaviors are facilitators of a wide range of desirable student-related academic outcomes. Then, to support our argument, we provide empirical evidence. In doing so, we introduce and define seven instances of positive teacher interpersonal communication behaviors, namely teacher care, clarity, credibility, rapport with students, stroke, immediacy, and confirmation, and expound how they positively predict academic outcomes such as motivation, learning, engagement, involvement, class attendance, willingness to communicate, performance, and success in students. Subsequently, we highlight the critical role of teacher interpersonal variables in the foreign/second language classroom context. Next, we suggest some pedagogical implications with the potential to enlighten the practice of key educational stakeholders (i.e., teachers, students, teacher educators, materials developers, administrators, and teacher recruiters). At the end, the limitations in this line of research are identified, and avenues for future research on teacher interpersonal communication in both general education and language education domains are put forward for interested researchers.

## Introduction

The recent special issue in Frontiers in Psychology, entitled “*The Role of Teacher Interpersonal Variables in Students’ Academic Engagement, Success, and Motivation*” clearly indicates its Editors’ concern with highlighting the importance of attending to teacher interpersonal communication behaviors and the immediate need to promote research in this line of inquiry. As a response to this call, the present conceptual review article endeavors to introduce what positive teacher interpersonal communication behaviors are, which theories underpin them, what significance they have for students’ academic practices, how crucial they are in both general education and language education, how this line of research can enlighten the practice of key stakeholders in the educational context, and finally, which aspects of instructional communication research require more empirical evidence.

Since the time of Plato and Socrates, teacher–student connection and the outcomes associated with that connection have been the focus of much research (Violanti et al., 2018), and it has been rather unanimously found that positive teacher–student interpersonal relationships are strong facilitators of a wide range of desirable student-related outcomes including engagement, learning, achievement, well-being, motivation, success, and hope, among others (Wendt and Courduff, 2018; Derakhshan et al., 2019; Frymier et al., 2019; Havik and Westergård, 2019; Pishghadam et al., 2019, 2021; Derakhshan, 2021). This is because teaching is essentially a relational profession. McIntyre et al. (2020) confirm that “teachers make great impact … in every moment of classroom learning” and “teachers’ moment-to-moment behaviors create an ever-evolving picture of who the teacher is” (p. 1).

The relationship between students and teachers is important because both are equally in charge of the successful realization of the instructional and learning processes (Delos Reyes and Torio, 2020). Hence, they must work together to build desirable learning conditions. Instructors stimulate the establishment of such conditions through employing relational behaviors that are associated with students’ positive experiences (Bolkan et al., 2015). It can be stated that learning involves more than just mere exposure to information; rather, it encompasses social, psychological, and emotional interactions. Therefore, effective instruction is usually actualized within the positive teacher–student relationship context (Strachan, 2020). Despite the fact that teacher–student relationships are integral aspects of any learning environment, the process of creating and maintaining a positive interpersonal relationship is a demanding task even for many experienced teachers (Strachan, 2020). Therefore, understanding the processes underlying effective teacher–student relationships is of utmost significance.

A positive instructor–student relationship is identified with empathy, caring, involvement, trust, and respect. It is theorized that, in relational terms, for enhancing students’ deep engagement with teachers, teachers should be approachable, believe in all their students, be empathetic, be responsive to students’ individuality, support students’ autonomy, and be passionate about their profession (Frisby, 2019; Mercer and Dörnyei, 2020). For these things to happen, teachers can take different actions such as taking care of their talk, being careful about feedback to students, listening to learners, employing questions to engage students, and rethinking classroom management as managing relationships (Mercer and Dörnyei, 2020). Claus et al. (2012) proclaim that when an intimate, positive teacher–student relationship is present, students and instructors initiate “meeting each other, learning about one another, developing expectations, and focusing on achieving goals” (p. 167).

Positive teacher interpersonal communication behaviors can be either verbal or non-verbal. Teacher care, stroke, immediacy, credibility, immediacy, clarity, confirmation, relational closeness to students, humor, and praise are all instances of teacher positive communication behaviors studied so far by researchers (Frisby, 2019). All these behaviors promote effective teacher–student communication, result in classroom vitality, and satisfy learners’ needs for emotional and interpersonal support (Goldman et al., 2017). Put it simply, these behaviors fulfill students’ relational, rhetorical, and emotional needs and wants (Frymier, 2016).

Positive teacher communication can be explained in light of positive psychology which has attracted much attention during the two last decades (Seligman, 2018), encompassing three main pillars: (1) positive experiences, (2) positive individual traits, and (3) positive institutions. It is assumed that when productive interactions exist between students and instructors, and a friendly and desirable classroom climate is present, students are more likely to experience positive emotions which are at the heart of successful teaching and learning (Seligman and Csikszentmihalyi, 2000). Positive psychologists have endeavored to uncover how individuals can prosper in more positive and favorable conditions. Consequently, it can be stated that positive psychology has brought about a major shift in the focus of psychology, from the obsession with only negative and undesirable events and behaviors in life toward more positive qualities (Seligman, 2011).

Teacher positive interpersonal communication can be also grounded in the rhetorical and relational goal theory (Mottet et al., 2006). The relational perspective toward instruction accentuates the quality of teacher–student relationships and the necessary skills to create and keep a good relationship in the instructional context (Rudick and Golsan, 2014). This theory is based on six assumptions; first, learners have both relational and academic wants; second, teachers have both rhetorical and relational goals; third, successful teaching is the result of specifying appropriate rhetorical and relational goals and utilizing suitable communication behaviors to accomplish those goals; fourth, learners who feel more content in the classroom and whose relational and academic needs are fulfilled, feel more motivated to learn, less disengaged, and more accomplishment; fifth, what goals instructors have and how they accomplish those goals is different across grade levels and contexts; and sixth, students at different stages of development have different relational and academic wants and the fulfillment of these wants and needs differ across stages of development and contexts (Houser and Hosek, 2018). Based on this theory, it can be concluded that when instructors utilize efficient interpersonal communication cues to meet learners’ relational and rhetorical wants, learners are more likely to experience a wide range of desirable outcomes including learning, interest, engagement, empowerment, motivation, and achievement (Houser and Hosek, 2018).

Research evidence has corroborated that teachers who provide more emotionally supportive classroom interactions are normally perceived by their students to be more just and caring (Gasser et al., 2018). The importance of teacher positive interpersonal treatment of students is also reflected in the concept of Loving Pedagogy. It is believed that “pedagogical love is oriented toward students’ needs,” the satisfaction of which demands teachers to be respectful, caring, understanding, and sensitive toward students (Yin et al., 2019). Therefore, one of the main pillars of a loving pedagogy is a loving teacher who is competent at nourishing students’ emotional, interpersonal, affective, and academic potentials (Yin et al., 2019).

Due to space constraints, in what follows, we succinctly touch upon seven key positive teacher communication behaviors, provide concise definitions for them, and report student-related outcomes empirically proved to be predicted by these communication behaviors.

### Teacher Care

Noddings (1984) first introduced the concept of care, reflected in senses of compassion, openness to the needs of others, closeness, and empathy toward others in interactions, relations, and encounters of a caregiver with a person being the receiver of the care (Meyers et al., 2019). In the instructional context, teacher care toward students represents a significant aspect of teacher–student relationships (Gasser et al., 2018). Teacher care pertains to teachers’ provision of genuine support to students, displaying interest in students’ learning, and being empathetic toward them (Gabryś-Barker, 2016). Teacher care refers to teachers’ behaviors to satisfy learners’ psychological and emotional needs by providing a respectful, positive, supportive, and nourishing environment (Laletas and Reupert, 2016). Research has consistently indicated that teacher emotional support for students improves the student–teacher relationship quality (Gasser et al., 2018). Similarly, from a theoretical vantage point (Noddings, 2006), teacher care is conceptualized as a crucial component of establishing and sustaining quality teacher–student relationships. Laletas and Reupert (2016) consider teacher care as so essential that they maintain care is an integral lynchpin of both discipline strategy and pedagogy. It is assumed that when students are aware of and feel teachers’ caring toward themselves, they feel secure and experience its positive consequences (Noddings, 2006). Teacher care stimulates student-related experiences like engagement, self-esteem, well-being, feeling respected, engagement, and performance (Derakhshan et al., 2019; Havik and Westergård, 2019).

### Teacher Clarity

Clarity is conceived as a process whereby the instructor and students communicate and negotiate meaning to make information more understandable (Myers et al., 2014). Within this process, teacher clarity behaviors refer to the instructor’s use of (non)verbal messages and cues such as underscoring main ideas, rewording main ideas, providing examples, illustrations, and visuals, and repeating main points to ease students’ comprehension, understanding, and final attainment (Violanti et al., 2018). At its operationalized level, teacher clarity is defined as a high-inference variable involving students’ perceptions regarding their instructors’ use of clarity behaviors to teach more transparently. The concept of teacher clarity is grounded in the theories of information processing and adaptive instruction. According to information processing, students are regarded as information processors and instructors are considered information dispensers (Segabutla and Evans, 2019). Students transfer the input they receive to the short-term memory, where some mental operations are applied to the information to be prepared for transference to the long-term memory (Bolkan, 2017). Clarity behaviors that teachers employ better help learners to go through the stages of processing, storing, and retrieving information (Titsworth et al., 2015). Regarding adaptive instruction, it is assumed that instructors are required to adapt their clarity behaviors to learners by means of communication. This clarity happens in the classroom when learners and instructors negotiate meaning during classroom communications. In this process, instructors prepare and present information, learners respond, give comments, and ask questions, and instructors respond when necessary to improve understanding (Bolkan, 2017). Teacher clarity is a rhetorical instructional behavior that positively influences learners’ outcomes, including learning (Titsworth et al., 2015; Violanti et al., 2018), affect for the course and teacher, motivation (Bolkan et al., 2015), understanding, empowerment (Finn and Schrodt, 2012), and engagement (BrckaLorenz et al., 2012).

### Teacher Confirmation

Interpersonal communication is conceived as a two-edged sword as it can confirm and build us up, or disconfirm or tear us down. Disconfirming and confirming responses enable us to establish a communication atmosphere, creating the emotional tie of interlocutors (Goldman et al., 2014). Through confirming communication, individuals feel endorsed, acknowledged, and recognized (Ellis, 2000). Thus, teacher confirmation pertains to teachers’ communicative attempts to convey to students that they are valuable (Burns et al., 2017). To achieve this goal, teachers typically avoid disconfirming students, answer students’ questions and provide them with feedback, show enthusiasm in students’ learning, and engage in an interactive teaching style (Ellis, 2000; Goldman et al., 2014). When teachers show confirmation of their students, they are involved in creating enjoyable instructional and learning environments (Edwards et al., 2011). Students need to be confirmed by their teachers, and teachers can do so by attending to what students say, think, or feel, indicating their recognition of students’ presence, and accepting the credibility of students’ thoughts and feelings; as a result, students feel more significant (Buber, 1957). Research has approved that when teachers are confirming students, students’ learning and motivation are promoted, their effort and interest are enhanced (Campbell et al., 2009), students feel more satisfaction (Goodboy et al., 2009), show more willingness to talk, feel to be better prepared and more involved (Sidelinger and Booth-Butterfield, 2010), and perceive the course as valuable (Horan et al., 2011). Teacher confirmation can also predict students’ emotional outcomes (Goldman et al., 2014), success, engagement, understanding, learning (Hsu, 2012), and communicative behaviors (Johnson and LaBelle, 2020). Compared to other teacher interpersonal behaviors, teacher confirmation has been the focus of less research.

### Teacher Credibility

Aristotle categorized modes of persuasion into Logos (the rationale employed to substantiate a claim), Pathos (the motivational and affective appeal), and Ethos (credibility of the speaker), all assumed to be influential in affecting the receiver of a message. The Ethos; that is the speaker’s being credible, is found to increase the effectiveness of communication (Pishghadam et al., 2017). More particularly, in the domain of education, classroom is conceived as a persuasive context, and the instructor is the one to persuade the learners (Gray et al., 2011). In this respect, teacher credibility pertains to students’ perceptions of the extent that their teacher is trustworthy, credible, or believable. Teven and McCroskey (1997) argued that teacher credibility involves three dimensions of goodwill, competence, and trustworthiness. Empirical studies in the domain of general education, language education, and communication education have approved the predictive role of teacher credibility for a wide range of student-related outcomes such as willingness to attend classes (Pishghadam et al., 2019, 2021), foreign language achievement (Pishghadam et al., 2017), motivation, learning (Gray et al., 2011), and engagement (Derakhshan, 2021).

### Teacher Immediacy

As a crucial component of effective communication (Finn and Schrodt, 2012), immediacy was introduced by Mehrabian (1967) as behaviors communicating interpersonal closeness and approachability. Within the instructional context, teacher immediacy is defined as verbal and non-verbal cues decreasing teacher–student physical or/and psychological distance (Estepp and Roberts, 2015). Teacher immediacy facilitates students’ needs satisfaction (Frymier, 2016). Verbal immediacy behaviors include engaging in friendly conversation with students, asking about students’ opinions, and using humor, while non-verbal immediacy cues include having a relaxed posture, leaning forward, having appropriate eye-contact, and smiling to students (Wendt and Courduff, 2018; Derakhshan, 2021). Such immediacy cues promote positive feelings and greatly facilitate effective instruction (Hampton, 2018). Compared to other teacher communication behaviors, immediacy is a more investigated concept. Immediacy was found to be a positive predictor of a wide range of student experiences including online engagement (Dixson et al., 2017), learning (Violanti et al., 2018), reduced foreign language anxiety (Ballester, 2015), motivation (Frymier et al., 2019), and academic engagement (Estepp and Roberts, 2015; Derakhshan, 2021).

### Teacher Stroke

Berne’s Transactional Analysis theory is a theory of systematic therapy and personality, explaining individuals’ personal change and growth and with fruitful implications for developing positive instructor–student relationships (Berne, 1988). Stroke is one of the elements of the transactional analysis theory, defined as one’s attempts to display attention to others’ hunger for recognition (Pishghadam and Khajavy, 2014). In the educational context, the teacher is the stroker (i.e., the person who gives stroke), and the student is the strokee (i.e., the one receiving stroke). Strokes can be positive (e.g., you look beautiful) or negative (e.g., I hate you); verbal (e.g., saying goodbye) or non-verbal (e.g., smiling, nodding); and conditional (e.g., you are a good student) or unconditional (e.g., I love you). In essence, people seek stroke and are strokable; therefore, when stroke is not present, individuals perceive being deprived. It is believed that even providing negative stroke is better than not providing any stroke (Derakhshan et al., 2019). Compared to other teacher positive interpersonal communication variables, teacher stroke is an under-researched topic. Previous studies have shown that teacher stroke is positively associated with teacher factors such as teacher credibility, success (Pishghadam et al., 2019, 2021), care, conceptions of intelligence (Derakhshan et al., 2019) as well as student factors such as motivation (Pishghadam and Khajavy, 2014), willingness to attend classes (Pishghadam et al., 2019, 2021), and foreign language achievement (Rajabnejad et al., 2017).

### Teacher–Student Rapport

Rapport refers to a harmonious teacher–student relationship (Delos Reyes and Torio, 2020), identified with enjoyment, connection, respect, and mutual trust (Frisby and Housley Gaffney, 2015). Rapport is an interpersonal bond during the teaching process which is greatly relationship-based (Frisby and Martin, 2010). Compared to other instructional communication variables, rapport is less investigated (Frisby et al., 2016). Yet, it is one of the most crucial elements of instructional communication as student learning initiates from rapport (Wilson et al., 2010), and rapport is an inseparable aspect of education. Teachers can establish rapport in the classroom through promoting free expression, respecting students’ attitudes, giving appropriate feedback, using humor, showing enthusiasm in students’ learning, and being gentle and optimistic (Weimer, 2010). Rapport also brings about positive experiences for students, including greater classroom participation, motivation (Estepp and Roberts, 2015; Frisby et al., 2016), peer-to-peer connectedness, learning (Frisby and Martin, 2010; Frisby, 2019), grades (Wilson and Ryan, 2013), engagement (Culpeper and Kan, 2020), as well as autonomy and achievement (Delos Reyes and Torio, 2020).

**FIGURE 1:**
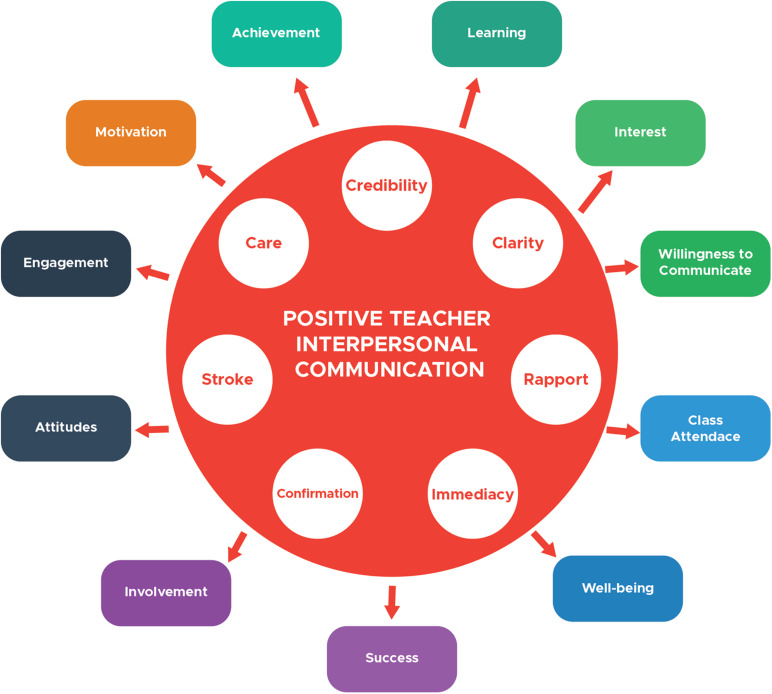
The schematic representation of the role of positive teacher interpersonal communication Behaviors in positive student-related academic outcomes.

All in all, the empirical evidence on the role of all the mentioned positive teacher interpersonal communication behaviors in promoting student-related positive outcomes is well justified by the rhetorical and relational goal theory in the instructional communication research (Mottet et al., 2006). Accordingly, when teachers specify rhetorical and relational goals and use proper verbal and non-verbal communication behaviors to simultaneously accomplish their own goals and satisfy learners’ needs, negative academic outcomes mitigate while positive outcomes are promoted (Houser and Hosek, 2018). Figure 1 portrays the schematic representation of what has been argued so far regarding the relationships of positive teacher interpersonal communication behaviors and student-related outcomes.

## Positive Teacher Interpersonal Communication in the Foreign/Second Language Classroom

Four decades of research in SLA has mainly focused on teacher and students’ negative emotions and outcomes by studying factors like anxiety, disengagement, burnout, depression, resistance, and stress (e.g., Gkonou et al., 2017; Seifalian and Derakhshan, 2018; Fathi and Derakhshan, 2019). However, with their recent advent of positive psychology and the call for its application in SLA by leading scholars (e.g., Mercer and MacIntyre, 2014; Mercer et al., 2018; MacIntyre et al., 2019; Budzińska and Majchrzak, 2021), SLA researchers have shifted their attention to the more bright side of the issue by initiating the study of positive language teachers and students’ emotions, behaviors, and outcomes (Dewaele et al., 2019; Bielak and Mystkowska-Wiertelak, 2020; Fathi et al., 2020; Greenier et al., 2021). Benesch (2017) maintained that L2 classes are filled with both negative and positive emotions; the former impeding successful teaching and learning and the latter fostering them. Furthermore, since language instruction and learning are inherently interactional, they require the integration of personally meaningful content and identities which are facilitated through teachers’ interpersonal and emotional understandings of learners.

In his book, entitled “*Positive Psychology Perspectives on Foreign Language Learning and Teaching*,” Gabryś-Barker (2016) stated that positive emotions, students’ personality traits, and learning environments, are the three main elements of L2 learners’ academic performance. It is believed that when a close interpersonal bond exists between the teacher and students and a relationship of trust is formed between them, a more favorable language learning classroom environment is created, and students’ foreign language enjoyment is facilitated. It is found that when students experience higher levels of enjoyment, their foreign language proficiency, performance, achievement, and willingness to communicate (Oxford, 2016; Dewaele et al., 2017; Mahmoodzadeh and Khajavy, 2019; Wei et al., 2019) are boosted. Khajavy et al. (2018) approve this argument by stating that the existence of a positive classroom environment and positive emotions in language classes concurrently mitigate L2 learners’ anxiety and increase their enjoyment and willingness to communicate.

The quality of teacher–student relationship is quite important in the L2 context (Mercer and Gkonou, 2020) because language learning is an inherently social process, much more than other academic subjects. The knowledge of language is typically learned and employed effectively through different modes of communication (Frymier et al., 2019). Thus, the requirement for interactions with fellow interlocutors (i.e., the teacher or peers), is highly felt. How well teachers and students get on with each other can make or break their teaching and learning experiences, respectively. The key relationship in education for both instructors and students is that between students and instructors, which highlights the important role of language teachers in preparing the floor for such relationships (Mercer and Dörnyei, 2020).

As rightly posited by Dewaele et al. (2017), the role of the L2 teacher is not just constrained to the transmission of linguistic and content knowledge to L2 learners. But more importantly, L2 teachers are held responsible for providing a positive environment, managing the emotional atmosphere of the classroom, establishing a good rapport with learners, and ideally, instructing with passion and joy. Therefore, L2 teachers’ positive attitudes, recognition and appreciation of students, and support for them are all instances of teacher interpersonal communication cues that might be perceived as lynchpins to L2 students’ desirable academic outcomes and experiences (Li et al., 2018).

In the same vein, it is argued that classroom interactions greatly influence foreign language enjoyment. Positive classroom interactions happen through supportive and friendly peer relationships as well as positive and encouraging behaviors of teachers toward students (Pavelescu and Petric, 2018; Pishghadam et al., 2021). It seems that two factors play influential parts in foreign language enjoyment; one is the classroom atmosphere (e.g., positive engagement, positive atmosphere, and peer interaction), and the other is the teacher (e.g., teacher understanding, care, recognition, attention, and positive attitude) (Mercer and Dörnyei, 2020) greatly highlighting the role of teacher interpersonal treatment of students in the language classroom (Li et al., 2018). When effective teacher–student relationships are formed, desirable student-related outcomes such as L2 motivation (Henry and Thorsen, 2018), L2 learning gains (Sánchez et al., 2013), and L2 engagement (Mercer and Dörnyei, 2020) are around the corner.

## Discussion

So far, we described: (1) what positive teacher interpersonal communication behaviors are, (2) which theories (i.e., positive psychology and the rhetorical and relational goal theory) underpin them, (3) seven instances of positive teacher interpersonal communication behaviors (i.e., teacher care, immediacy, stroke, credibility, rapport with students, stroke, and confirmation) and their contributions to desirable student-related outcomes like motivation, engagement, success, and learning, and (4) the significance of positive teacher communication behaviors in the foreign/second language classroom. Based on what was conceptually reviewed, it seems that this area of research pedagogically contributes to the field by informing the practice of key educational stakeholders like school principals, educational supervisors, teacher recruiting committees, materials developers, teacher educators, pre- and in-service teachers, and students.

For instance, those authorities in charge of recruiting effective instructors should become aware that the responsibilities of teachers are not limited to the transmission of content and pedagogical knowledge. Rather, teachers are held responsible for making effective interpersonal relationships, creating bonds of trust between themselves and students, and building an enjoyable learning environment. Accordingly, these stakeholders must revisit and expand standards for qualifying effective teachers by considering teachers’ relational and affective treatment of students as a required criterion for teachers to enter the education system. Similarly, school managers and supervisors who are in charge of constantly evaluating the effectiveness of teachers who have entered the education system can benefit from research evidence in the domain of instructional communication through engaging in such activities as observing teachers’ actual interpersonal practices in the classroom or interviewing their teachers to gage their knowledge of teacher interpersonal communication and its significance for students’ academic performance.

This area of research can also be redound to the benefit of teacher educators and trainers responsible for holding workshops, teacher education programs, and teacher training courses for pre- and in-service teachers. Unfortunately, these interventional programs are obsessed with building teachers’ pedagogical and content knowledge to the disregard of other neglected but equally important aspects of being an effective teacher including teachers’ ability to have effective interpersonal communication with students (Derakhshan et al., 2020a). This teacher characteristic can be built through such behaviors as caring for students, respecting students’ attitudes, providing appropriate feedback regarding their performance, confirming students’ presence and importance, and building a relationship of trust between themselves and their students. Therefore, teacher educators can reduce the gap between theory and practice in instructional communication by directly teaching teacher attendees regarding teacher interpersonal communication behaviors, the theories behind them, their contribution to students’ practices, and the ways they can enact relational goals in the classroom. Such training workshops and programs can be divided into two parts; the first being conceptual, being concerned with familiarizing teachers with the main concepts in instructional communication, and the second being related to teachers’ actual practice of what they have learned in the first part of the program.

Moreover, materials developers can benefit from this line of research by taking them into account when designing teacher books, student textbooks, workbooks, and supplementary books. In this regard, materials developers are expected to consider successful teacher–student interpersonal relationships as a main element of learning and teaching when designing reading texts, tasks, activities, questions, and exercises. For instance, when designing tasks in textbooks, materials developers can write them in a way promoting peer and teacher–student discussions and reaching rapport to successfully accomplish a learning task. Last but not least, teachers can increase their effectiveness by continuously updating their knowledge repertoire with recent research evidence in instructional communication, reflecting on their relational practices in the classroom, engaging in constant evaluation of their interpersonal treatment of students both during and after each session of classes, engaging in discussion with students in and out of class to better discover their students’ relational and academic needs and accordingly finding the most effective teaching and relational practices that best suit a group of students and fulfill their needs.

All in all, the review of the literature on the role of positive teacher interpersonal variables in student-related outcomes revealed some limitations in the studies done in this area. To start with, it should be stated that different teacher interpersonal variables have not been equally researched; for instance, compared to other interpersonal instances, teacher immediacy has been the focus of much research (e.g., Dixson et al., 2017; Violanti et al., 2018; Wendt and Courduff, 2018; Derakhshan, 2021), while other interpersonal variables like teacher confirmation and stroke have been less investigated (e.g., Campbell et al., 2009; Sidelinger and Booth-Butterfield, 2010; Hsu, 2012; Pishghadam et al., 2019, 2021). As it is argued that all instances of teacher interpersonal communication behaviors contribute to successful teacher–student relationships and promote desirable student outcomes (Houser and Hosek, 2018), it is necessary that all of them be studied across different contexts, grade levels, and learners’ stages of development to see how they converge or diverge with regard to their effects on students’ outcomes. Some future studies can also simultaneously examine two or more teacher interpersonal variables in relation to a specific student outcome in a single study to unravel the inter-relationships of the studied interpersonal variables and uncover the extent to which each of them can predict student desirable experiences.

Next, the majority of the studies have been quantitative, engaging in one-shot study of their variables mostly in survey studies (e.g., Estepp and Roberts, 2015; Finn and Schrodt, 2016; Frymier et al., 2019; Havik and Westergård, 2019). Thus, future researchers are recommended to shift their attention to more qualitative or mixed-methods research approaches which can potentially engage in more detailed and deeper understanding of an issue under investigation. In this regard, researchers are recommended to do more longitudinal studies which can show how an issue changes over time. Researchers can also conduct case studies by focusing on perceptions, attitudes, or experiences of a few selected cases and reaching rich data about them. Furthermore, the main instrument used in the studies has been a questionnaire. In this regard, future researchers can also use other instruments like interviews, observation schemes, diary writing, journal, field note, and documentation.

Research evidence (e.g., McCroskey and McCroskey, 2006) evinces that the majority of the studies have been conducted in the United States with a mainly Anglo-European culture. To address this notion, McCroskey and McCroskey (2006) called researchers to engage in culture-centered instructional communication research. Some researchers answered this call by studying teacher interpersonal communication in cultures like Japan (e.g., Zhang et al., 2007), Brazil (Santilli et al., 2011), Turkey (Frisby et al., 2016), Iran, Iraq (e.g., Derakhshan, 2021; Pishghadam et al., 2021), South Korea (Mansson and Lee, 2014), and Germany (Zhang et al., 2007). However, instructional communication issues of other cultures are still under-researched. The paucity of research in this area demands utmost attention by researchers to make cross-cultural comparisons and replicate accepted lines of research in diverse cultures and as a result, logically extend established theories.

As students and teachers’ mindset is shaped by their cultural backgrounds, there is a need to understand the extent to which teacher interpersonal communication behaviors are perceived, acted out, and experienced similarly or dissimilarly across cultures. This argument can also be supported by the fifth and sixth tenets of the rhetorical and relational goal theory which posits that teachers’ rhetorical and relational goals and students’ academic and relational needs vary across contexts and age levels, and how those needs and goals are fulfilled and achieved also vary across contexts (Houser and Hosek, 2018) which pinpoint the significance of studying these issues in different geographical locations and cultural contexts.

Another lacuna in this area is that while teacher–students interpersonal relationships have been much investigated in general education, they are rather unattended to in the L2 context (Hagenauer and Volet, 2014). Therefore, due to the inherent interpersonal nature of language education (Mercer and Dörnyei, 2020) and following the recent emergence and burgeoning of positive psychology in SLA accentuating that positive emotions, students’ personality traits, and learning environments are the three main elements of L2 learners’ performance (Gabryś-Barker, 2016), it is hoped that more attention be paid to positive personal, psychological, emotional, or interpersonal aspects of L2 teaching and learning.

Additionally, the majority of the studies have focused on students’ perceptions and experiences of teacher interpersonal behaviors and their own educational outcomes to the neglect of teachers’ perceptions and experiences. Besides, as teachers and students are both playing a crucial role in successful learning and teaching and both contribute to the effectiveness of teacher–student relationships, the mere exploration of students’ perceptions do not provide us with a clear picture of what happens during the relational, learning, and instructional processes (Delos Reyes and Torio, 2020). Thus, future studies in this domain can investigate teachers’ perspectives. More importantly, as recommended by Derakhshan et al. (2020b), some researchers can concurrently study a teacher’s interpersonal communication issue from the perspective of both teachers and students to see how similar or dissimilar students and teachers might perceive or experience emotions, behaviors, and feelings in the same instructional context.

In the same vein, there is a shortage of studies on how pre- and in-service teachers’ interpersonal communication practices can be enhanced. To address this gap, future researchers can do experimental studies by providing a group of teachers with intervention on a particular aspect of interpersonal communication and check how receiving instruction can promote teachers’ interpersonal treatment of students. What all these research lacunas evince is that teacher interpersonal communication is a vast avenue for research, and there is still a large way to go to study all dimensions of this line of research. Thus, as a fertile area of research, instructional communication welcomes researchers worldwide to add to the body of literature in this area by studying its less-investigated aspects in the near future.

## Author Contributions

By drawing on the positive psychology movement and the rhetorical and relational goal theory in instructional communication, we argue that positive teacher interpersonal communication behaviors are facilitators of a wide range of desirable student-related academic outcomes. Then, to support our argument, we provide empirical evidence. In doing so, we introduce and define seven instances of positive teacher interpersonal communication behaviors, namely teacher care, clarity, credibility, rapport with students, stroke, immediacy, and confirmation, and expound how they positively predict academic outcomes such as motivation, learning, engagement, involvement, class attendance, willingness to communicate, performance, and success in students. Subsequently, we highlight the critical role of teacher interpersonal variables in the foreign/second language classroom context. Next, we suggest some pedagogical implications with the potential to enlighten the practice of key educational stakeholders (i.e., teachers, students, teacher educators, materials developers, administrators, and teacher recruiters).

## Conflict of Interest

The author declares that the research was conducted in the absence of any commercial or financial relationships that could be construed as a potential conflict of interest.
